# Surgical Treatment of Acute, Circumscribed, Pulmonary Gangrene; Second Successful Operation

**Published:** 1884-07

**Authors:** C. Fenger


### ARTICLE III.

### SURGICAL TREATMENT OF ACUTE, CIRCUMSCRIBED, PULMONARY GANGRENE; *Second Successful Operation; by* PROFESSOR C. FENGER, M. D.

Among the excellent voluntary papers presented at the last meeting of the Illinois State Medical Society, was a contribution to the surgical treatment of acute pulmonary gangrene, by Prof. Christian Fenger, M. D., of Chicago. The paper deserves and will elicit general attention, both from its intrinsic merit and as indicative of progress in American surgery.

As advised at the present time, four operations for acute pulmonary gangrene have been performed.

The first operation was performed in 1879, by Messrs. Lawson and Cayley, of England, in a case of pulmonary gangrene of five weeks' standing. Decided amelioration of symptoms, more particularly as regards cough, dyspnœa and fetor, was observed. The patient died of exhaustion four days after the operation. The autopsy disclosed facts which led the operators to believe that an earlier operation would have saved the patient's life. (Med. Society, London, 1880.)

Mr. Solomon Charles Smith, of Halifax, performed the second operation in 1880. The patient was in the second week of croupous pneumonia, when gangrene occurred in the lower lobe of the left lung. The patient lived ten days, with marked improvement in cough, dyspnœa and fetor. No autopsy was made.

Professor Buhl, of Christiana, performed the third operation in 1880. Acute gangrene in the anterior portion of the left lung was the indication for operation. After a long convalescence of six weeks the patient recovered.

The fourth operation was performed by Professor Christian

Fenger, of Chicago, in the Cook County Hospital, in April, 1884.

The patient, male, 34 years old, was in the second week of croupous pneumonia. Signs of consolidation and formation of a cavity in the right infra-mammary region, extending into the right axilla, were elicited by auscultation and percussion. Cough was distressing and dyspnœa great; about one pint of extremely offensive sputa was daily expectorated. The patient lost all appetite and rapidly progressive emaciation supervened. A cavity was found, upon the introduction of the needle of a hypodermic syringe through the thoracic wall, in the right infra-mammary region. An incision was made parallel to the clavicle; the ribs exsected to an extent sufficient to secure access to the part, and the needle re-introduced within the cavity, as a guide. The cavity was then cut down upon by the small platinum pole of a Paquelin's thermo-cautery, and an opening sufficient to admit the index finger secured. Digital exploration revealed no detached gangrenous masses. Accordingly, the cavity was gently washed out, a drainage tube inserted, and the usual antiseptic dressing applied. Hemorrhage during the operation was trifling, but washing out the cavity produced very troublesome coughing. The patient speedily reacted from the shock of the the operation, which was relatively slight.

Five hours after the operation, appreciable diminution in fetor was noted; at the end of the first week, expectoration was minimal, and fetor could not be perceived; at the end of the second week, decided improvement with return of appetite was observed; the fourth week witnessed further progress; and at the end of the fifth week the patient was out of bed. During convalescence bits of gangrenous lung tissue were discharged through the external opening.

With reference to the *technique* of the operation, Dr. Fenger recommends :—

1. The incision ought to be made parallel to the ribs.

2. The ribs must be exsected to a degree sufficient to secure access to the part.

3. In conformity with the suggestion of Albert and Mosler, the needle of a hypodermic syringe should be used as a guide into the cavity, or diseased lung tissue, and the small platinum pole of Paquelin's thermo-cautery should be employed to effect the opening.

4. The cavity must be washed out if practicable. Due care must be exercised to prevent drowning, if the cavity connects with a bronchus. Irrigation of the cavity was productive of no untoward effect in Buhl's case, but was the cause of troublesome cough in Fenger's patient, and Mosler ascribes one death to poisoning from thymol irrigation.

Dr. Fenger is of the opinion that there is no immediate danger of death from the operation, and that it is indicated in cases of acute, circumscribed, pulmonary gangrene.

